# Comparison of the Injury Mechanism between Pregnant and Non-Pregnant Women Vehicle Passengers Using Car Crash Test Dummies

**DOI:** 10.3390/healthcare10050884

**Published:** 2022-05-11

**Authors:** Ayumu Kuwahara, Masahito Hitosugi, Arisa Takeda, Seiji Tsujimura, Yasuhito Miyata

**Affiliations:** 1 Department of Legal Medicine, Shiga University of Medical Science, Otsu 520-2192, Japan; ds112129@g.shiga-med.ac.jp (A.K.); arisa204@belle.shiga-med.ac.jp (A.T.); 2 Joyson Safety Systems Japan K.K. Echigawa Plant, Otsu 529-1388, Japan; seiji.tsujimura@joysonsafety.com (S.T.); yasuhito.miyata@joysonsafety.com (Y.M.)

**Keywords:** pregnant women, frontal motor vehicle collisions, negative fetal outcome

## Abstract

This paper analyzes the kinematics and applied forces of pregnant and non-pregnant women dummies sitting in the rear seat during a frontal vehicle collision to determine differences in the features of abdominal injuries. Sled tests were conducted at 29 and 48 km/h with pregnant and non-pregnant dummies (i.e., MAMA IIB and Hybrid III). The overall kinematics of the dummy, resultant acceleration at the chest, transrational acceleration along each axis at the pelvis, and loads of the lap belt and shoulder belt were examined. The belt loads were higher for the MAMA IIB than for the Hybrid III because the MAMA IIB had a higher body mass than the Hybrid III. The differences in the lap belt loads were 1119 N at 29 km/h and 1981–2365 N at 48 km/h. Therefore, for restrained pregnant women sitting in the rear seat, stronger forces may apply to the lower abdomen during a high-velocity frontal collision. Our results suggest that for restrained pregnant women sitting in the rear seat, the severity of abdominal injuries and the risk of a negative fetal outcome depend on the collision velocity.

## 1. Introduction

Trauma is common among pregnant women, with 1 in 12 pregnancies affected [1]. Trauma is also the leading non-obstetric cause of death among reproductive-age women in the United States [2]. For pregnant women, adverse fetal outcomes, such as miscarriage, preterm labor, abruption of the placenta, and fetal demise, can occur. A systematic review of traumatic injuries among pregnant women revealed that injuries sustained in motor vehicle collisions (MVCs) were the most common and most life-threatening type of injury [3]. It has been estimated that approximately 160,000 pregnant women are involved in MVCs each year in the United States [4]. While most of these women suffer minor injuries, it has been estimated that 160 pregnant women are killed and an additional 600–2600 fetuses are killed when the mother survives [4,5,6,7,8]. Therefore, the highest priority worldwide in protecting both mothers and fetuses is preventing maternal injuries, especially MVC injuries. Generally, pregnant women involved in MVCs suffer from less severe injuries and have a lower fatality rate than non-pregnant women involved in MVCs. A retrospective cohort study using data from the Israel National Trauma Registry found that the severity of injury and the mortality rate among pregnant women involved in MVCs were significantly lower than those of non-pregnant women in MVCs [9]. A population-based matched retrospective cohort study using data from the Nationwide Inpatient Database in the United States suggested that pregnant women admitted to hospital following MVCs sustained less severe injuries than non-pregnant women [10].

Injuries sustained during pregnancy, when the lower abdomen protrudes according to the gestational age, have been considered different from those sustained before pregnancy. Several reports have suggested differences between pregnant and non-pregnant women in the type and severity of abdominal injuries due to the fact of MVCs. A retrospective analysis using data from the NASS/CDS database compared the injury severity between pregnant and non-pregnant women. This comparison was made for 1059 pregnant women and 34,439 non-pregnant women, and the backgrounds of the collisions were similar between the two groups. The study concluded that the risk of an abbreviated injury severity (AIS) score of two or more (AIS 2+) for injury to the abdomen was similar in the two groups [11]. However, a single-center retrospective observational study suggested that the AIS score for the abdomen was lower in pregnant women than in non-pregnant women. In the cited study, although a comparison was made by matching for age, seatbelt use, and deployment of an airbag, the position of the seat was not considered [12]. A nationwide collision data-based study comparing the detailed characteristics and outcomes of MVCs involving pregnant and non-pregnant women reported that the rate of AIS 2+ injuries of the abdomen was higher in pregnant women than in non-pregnant women. There were no significant differences in the background of the collisions such as the height of the woman, seatbelt use, direction of force, collision velocity, or the occurrence of rollover [13]. Therefore, there have been differences in the prevalence and severity of abdominal injuries between pregnant and non-pregnant women among studies based on crash data. As these differences may partly be due to the differences in the background of the collisions, the results need to be confirmed from another viewpoint. Analyses of the kinematics of pregnant and non-pregnant women are required to clarify the differences in features of abdominal injuries. However, there have been no reports on a difference in kinematics between pregnant and non-pregnant women.

The use of a mechanical model with high human similarity is appropriate to determine the kinematics of vehicle passengers. Although various types of dummies have been used in officially recognized automotive accreditation tests, only one pregnant woman dummy has been used (Maternal Anthropometric Measurement Apparatus dummy, version IIB; MAMA IIB) [14]. Studies have adopted the MAMA IIB to improve safety for pregnant women passengers [15,16,17,18,19,20,21,22,23]. In most of these studies, the dummy was sat on the driver’s seat, and its kinematics and applied physical parameters were obtained. However, a questionnaire survey of pregnant women revealed that women preferred the rear seat more during pregnancy (28.8%) than before pregnancy (21.0%) [24]. Studies on the safety of pregnant women sitting in rear seats are thus required. There has been only one study in which the MAMA IIB was sat on the rear seat and sled tests were performed [15]. However, this study examined neck injuries due to the fact of shoulder belt compression and not the kinematics or physical parameters of the abdomen. The objectives of the present study were, first, to confirm the kinematic differences of the trunk, especially for the abdomen, using pregnant and non-pregnant women dummies; second, to clarify the difference in abdominal injuries between pregnant and non-pregnant women.

## 2. Materials and Methods

The most recent version of the MAMA IIB, which was developed by First Technology Safety Systems and the University of Michigan Transportation Research Institute in 2001, was used [14]. This dummy was developed on the basis of the Hybrid III and is thus suitable for vehicle impact tests and for analyzing the kinematics of pregnant women during an MVC. The dummy has a modified pelvis and ribcage that allows the installation of a silicone rubber bladder representing the uterus at 30 weeks of gestation [25]. The size of the dummy is based on an American female dummy of the fifth percentile (AF05), which represents a small female with a height of 153 cm. This size is in accordance with a standard Japanese pregnant woman at 30 weeks of gestation [26]. We also used the Hybrid III-AF05 for comparison. Sled tests were performed at the HYGE sled test facility in accordance with previously published protocols [15].

In each test, the dummy was seated on the right side of the rear seat wearing a three-point seatbelt. The seat corresponded to a sedan type of mid-size car with a reclined torso angle of 20°. To represent situations of the frontal impact of a passenger vehicle, trapezoid waveforms measured in a flat barrier test with vector velocity changes at the time of impact (delta-V) of 29 and 48 km/h were applied to the sled.

One test using the MAMA IIB (test 1) and one test using the Hybrid III (test 2) were performed at a delta-V of 29 km/h. One test using the MAMAIIB (test 3) and two tests using the Hybrid III (tests 4 and 5) were performed at a delta-V of 48 km/h. The airbag and seatbelt pretensioner were not activated at any time throughout the tests. The overall kinematics of the dummy, such as trajectory during impact, were examined through high-speed video imaging. An accelerometer and load cell mounted on the dummy measured the resultant acceleration at the chest, transrational acceleration along each axis at the pelvis, and the loads of the lap belt and shoulder belt. Additionally, in the sled tests, forward displacement of the pelvis was measured. For comparison, the resultant acceleration was calculated as the square root of the sum of squares of each axial component of acceleration.

## 3. Results

### 3.1. Kinematics of the Dummy

**Figure 1:**
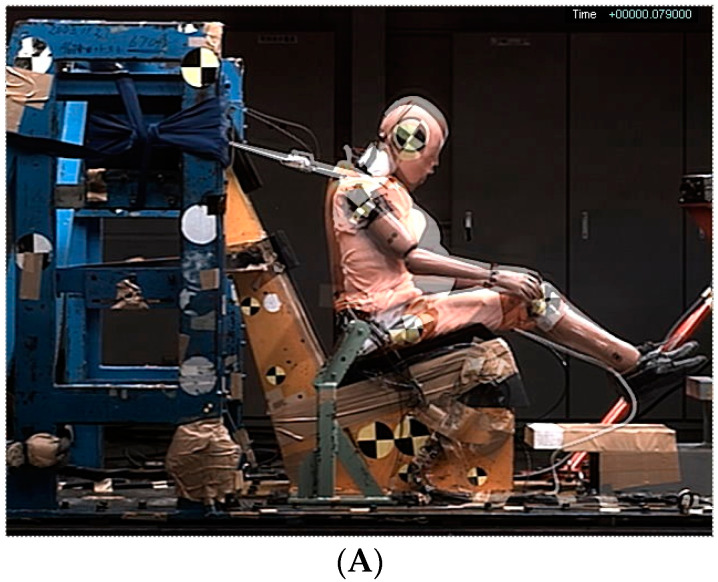

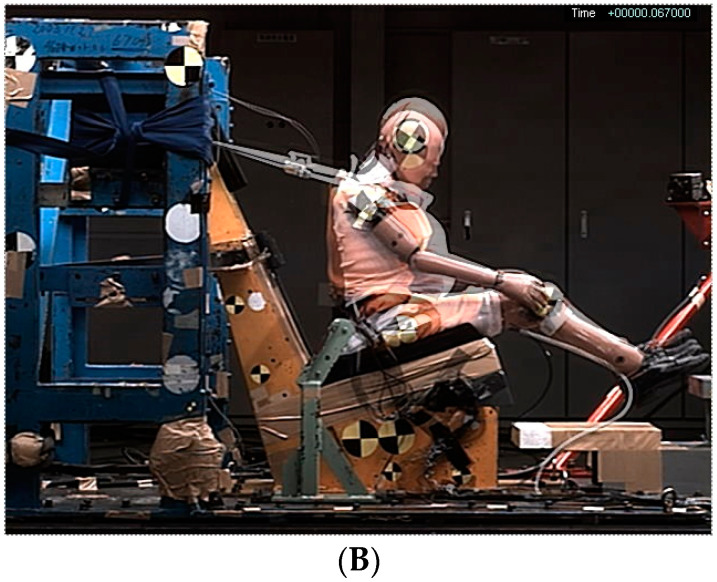
(**A**) Kinematics of the MAMA IIB and Hybrid III at a delta-V of 29 km/h in tests 1 and 2; (**B**) kinematics of the MAMA IIB and Hybrid III at a delta-V of 48 km/h in tests 3 and 4.

Figure 1A shows the kinematics of the dummy at 79 ms from the initiation of impact (onset of the sled pulse) taken by a high-speed video camera from the right side of the sled in tests 1 and 2. In the test using the. The MAMA IIB at a delta-V of 29 km/h (test 1), the dummy’s pelvis moved forward by 119.4 mm from its initial position at 79 ms from the initial impact (onset of the sled pulse), whereas in the test using the Hybrid III at a delta-V of 29 km/h (test 2), the dummy’s pelvis moved forward by 103.5 mm at 69 ms. Both the Hybrid III and MAMA IIB are shown in Figure 1A at 79 ms from the initial impact, with the shadow showing the MAMA IIB.

In the test using the MAMA IIB at a delta-V of 48 km/h (test 3), the dummy moved forward by 165.2 mm at 67 ms, and in the test using the Hybrid III at a delta-V of 48 km/h (tests 4 and 5), the dummy moved forward by 151.1 and 151.4 mm at 67 ms. According to tests 3 and 4, both dummies at 67 ms from initial impact are shown in Figure 1B.

### 3.2. Resultant Acceleration of the Chest

**Figure 2:**
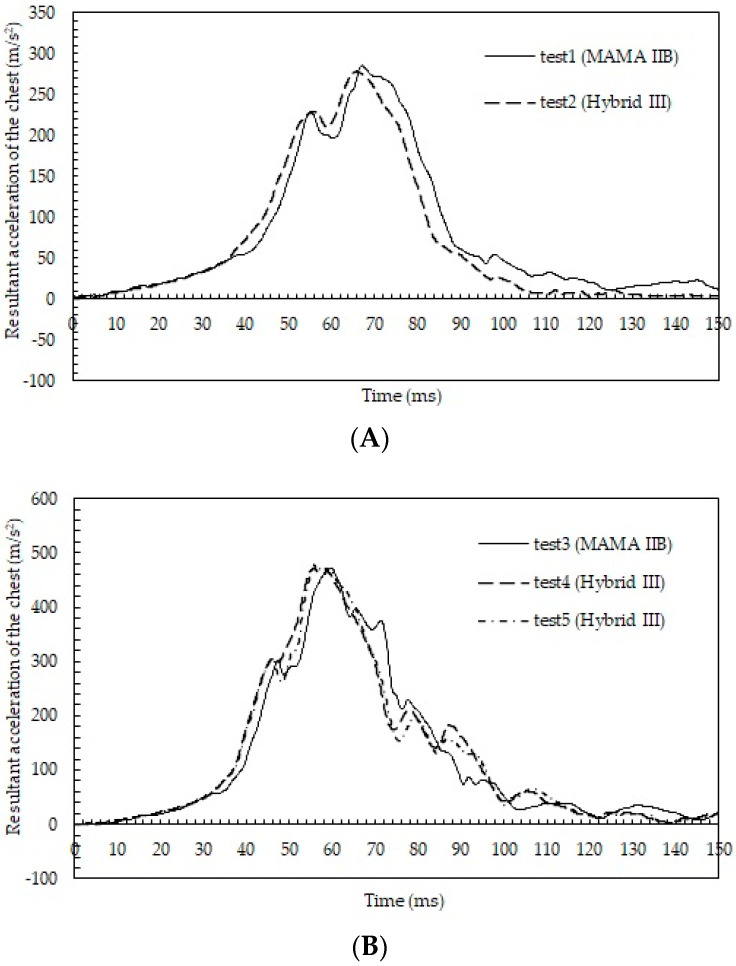
(**A**) Time courses of the resultant accelerations of the chest in tests 1 and 2; (**B**) time courses of the resultant accelerations of the chest in tests 3–5.

Figure 2A shows the time courses of the resultant chest accelerations in tests 1 and 2. Similar changes were obtained for the two dummies: the maximum acceleration was 285.5 m/s^2^ at 67.1 ms from the initiation of impact in test 1 and 278.3 m/s^2^ at 65.8 ms in test 2.

Figure 2B shows the time courses of the resultant chest accelerations in tests 3–5. The maximum resultant chest accelerations in tests 3–5 were 471.4 m/s^2^ at 59.6 ms, 471.1 m/s^2^ at 55.7 ms, and 478.1 m/s^2^ at 55.6 ms, and the curves of the acceleration were similar.

### 3.3. Longitudinal Acceleration of the Pelvis

**Figure 3:**
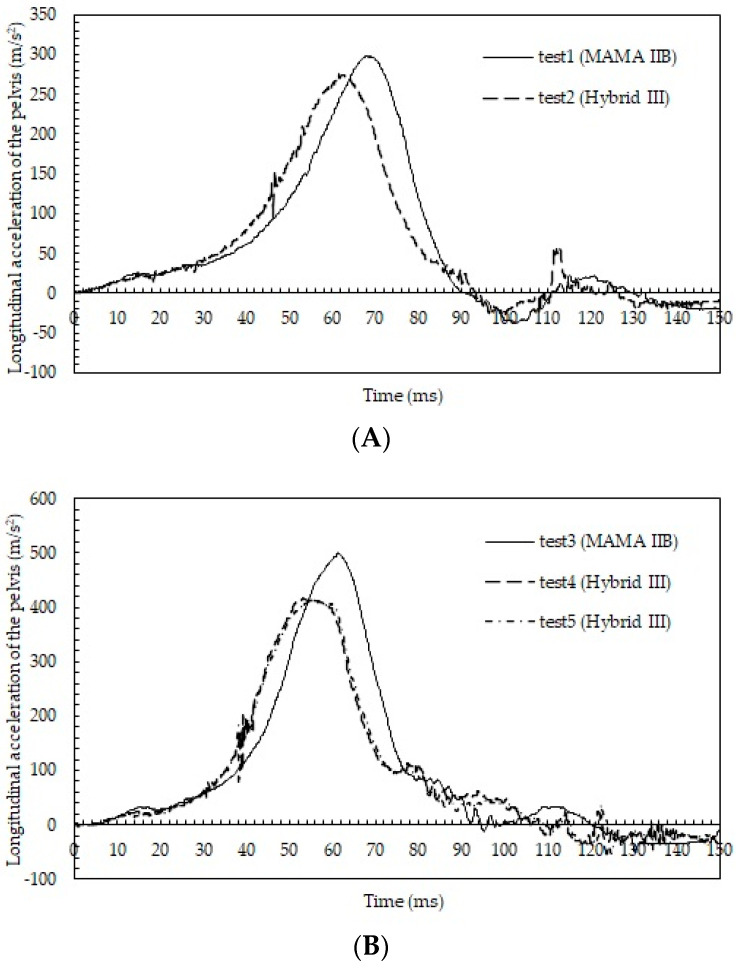
(**A**)Time courses of the longitudinal accelerations of the pelvis in tests 1 and 2; (**B**) time courses of the longitudinal accelerations of the pelvis in tests 3–5.

Time courses of the longitudinal accelerations of the pelvis are shown in Figure 3A for tests 1 and 2 and in Figure 3B for tests 3–5. At a delta-V of 29 km/h, the acceleration rose more rapidly for the Hybrid III than for the MAMA IIB; however, the maximum acceleration was higher for the MAMA IIB (298.2 m/s^2^) than for the Hybrid III (275.7 m/s^2^). At a delta-V of 48 km/h, the acceleration rose more rapidly for the Hybrid III (tests 4 and 5) than for the MAMA IIB (test 3). The maximum acceleration was higher for the MAMA IIB (500.4 m/s^2^) than for the Hybrid III (416.6 m/s^2^ in test 4 and 415.1 m/s^2^ in test 5).

### 3.4. Belt Loads

**Figure 4:**
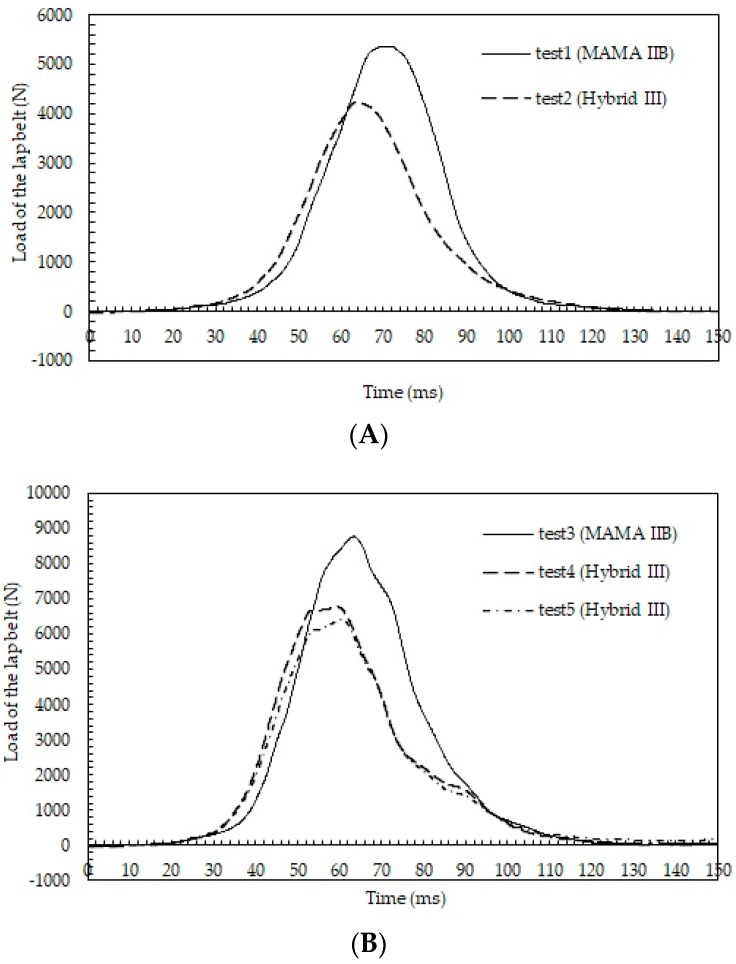
(**A**) Time courses of the loads of the lap belt in tests 1 and 2; (**B**) time courses of the loads of the lap belt in tests 3–5.

Time courses of the lap belt load are shown in Figure 4 (tests 1 and 2 in Figure 4A and tests 3–5 in Figure 4B). The belt loads increased more rapidly for the Hybrid III than for MAMA IIB. The maximum lap belt load was 5363 N for the MAMA IIB, higher than that for the Hybrid III (4244 N). Similar trends were observed in tests 3–5, with the maximum lap belt load of 8768 N for the MAMA IIB being higher than the maximum lap belt loads for the Hybrid III (6787 N in test 4 and 6403 N in test 5).

**Figure 5:**
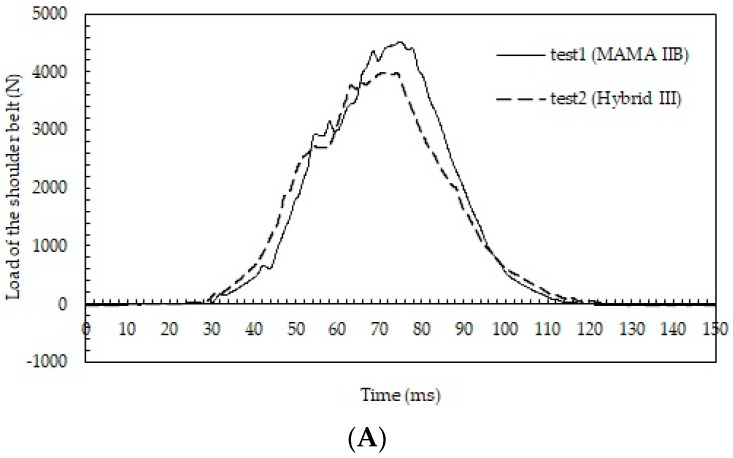

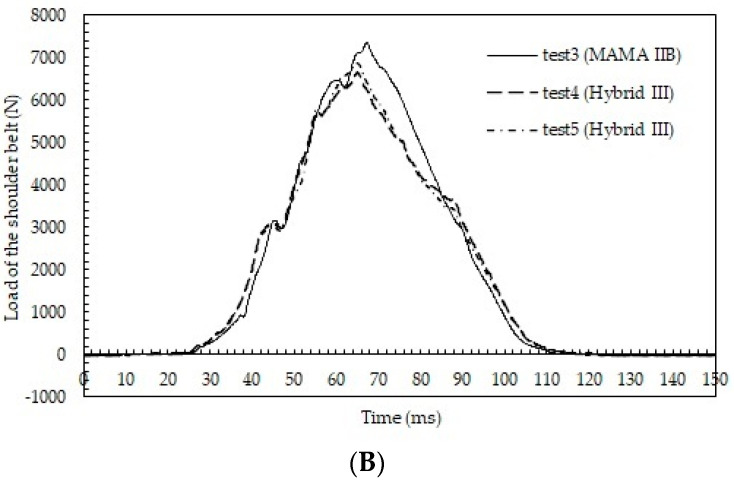
(**A**) Time courses of the loads of the shoulder belt in tests 1 and 2; (**B**) time courses of the loads of the shoulder belt in tests 3–5.

Time courses of the shoulder belt load are shown in Figure 5 (tests 1 and 2 in Figure 5A and tests 3–5 in Figure 5B). The maximum shoulder belt loads were higher for the MAMA IIB than for the Hybrid III at both vehicle collision velocities (i.e., 4524 N in test 1 and 3986 N in test 2; 7344 N in test 3, 6652 N in test 4, and 6866 N in test 5).

## 4. Discussion

In this study, we confirmed the kinematics and applied forces for pregnant and non-pregnant women sitting in the rear seat of a vehicle during a collision.

There was little difference in the distance moved forward between the MAMA IIB and Hybrid III at both 29 and 48 km/h for similar occupant restraint conditions. Therefore, for pregnant women, an effect of the seatbelt restriction similar to that for non-pregnant women was obtained. This result emphasizes the importance of correct seatbelt use for pregnant women. In 2001, before the legal enforcement of seatbelt use in the rear seat, the majority of pregnant women vehicle passengers (80.9%) never used a seatbelt in the rear seat in Japan [27]. After the revision of the road traffic law that legally required the use of a seatbelt in the rear seat in 2008, a study conducted in Japan suggested that 20.0% of pregnant women with a gestational age of 28 weeks or more always used a seatbelt, 20.2% often used a seatbelt, 27.0% sometimes used a seatbelt, and 32.8% never used a seatbelt [28]. Another report using NASS-CDS data revealed that the rate of seatbelt use in rear seats was lower than that in front seats [29]. Hence, we healthcare professionals have to contribute to improving the rate of correct seatbelt use, especially for pregnant women sitting in the rear seat. To improve the rate of seatbelt use, appropriate education, including presenting the scientific evidence concerning the benefit of wearing a seatbelt, is important, e.g., it can be communicated that for a given crash severity, an 84% reduction in the risk of adverse fetal outcomes can be achieved by properly wearing a seatbelt [30]. Thus, as previously suggested, healthcare professionals must provide education on correct seatbelt use early in pregnancy [31].

The results of the present study showed higher belt loads for the MAMA IIB than for the Hybrid III. The difference in the lap belt loads was approximately 1 kN at 29 km/h and approximately 2 kN at 48 km/h. These results were due to the difference in the dummies’ mass, with the MAMA IIB being 9 kg more massive than the Hybrid III. Additionally, the belt load increased more rapidly for the Hybrid III than for the MAMA IIB at both velocities. It is considered that the initial restraint of the lap belt was simply delayed for the MAMA IIB compared with the Hybrid III because of the difference in the shape of the abdomen, in that the lap belt was fitted to the lower abdomen of the non-pregnant dummy in contrast with the case for the pregnant dummy. However, as the distances of forward movement were similar for the two dummies in our study, the delay of the initial restraint of the lap belt for the MAMA IIB would be trivial.

In this study, the differences in the lap belt loads increased with the collision velocity. Therefore, for pregnant women sitting in rear seats, the lower abdomen may be subjected to stronger forces when there is a frontal collision at high vehicle velocity. A risk curve for an adverse fetal outcome based on real-world collisions involving pregnant women reveals that, even if using a seatbelt adequately, the risk increases with collision velocity in that the risk of an adverse fetal outcome exceeds 60% when the collision velocity exceeds 48 km/h [16]. This trend might be due to the forces acting on the abdomen via a lap belt, which agrees well with our results.

There are different results for the injury severity of the abdomen in the literature in that the risk of AIS 2+ injury has been shown to be higher, similar, or lower in pregnant women than in non-pregnant women [11,12,13]. Our results suggest that for the passenger restrained by a seatbelt, the risk depends on the collision velocity. At middle or lower velocities of collision, the risk of AIS 2+ injury may not increase in pregnant women; however, at a high velocity of collision, pregnant women may suffer stronger forces to the lower abdomen via a lap belt.

In this study, similar values for the acceleration of the chest were obtained for the two dummies. However, because the MAMA IIB had a greater body mass than the Hybrid III, shoulder belt loads were higher for the MAMA IIB than for the Hybrid III (7344 N vs. 6652 N and 6866 N at 48 km/h; 4524 N vs. 3986 N at 29 km/h). A previous study reconstructing a frontal vehicle collision using the MAMA IIB for the driver suggested that there was a shoulder belt load of 4.6 kN at a collision velocity of 40 km/h with the deployment of an airbag and activation of a pretensioner and force limiter [32]. Our result agrees well with this previous result. Additionally, the previous study considered the risk of a negative fetal outcome for restrained pregnant women in a frontal collision. As the chest deflection exceeded 28 mm at a collision velocity of 26 km/h or higher, a force may apply to the uterus because the uterus enlarges, and the fundus reaches the lower part of the chest in the late term of pregnancy [32]. Therefore, the present result suggests a substantial risk of a negative fetal outcome, even when using a seatbelt in the rear seat at a high velocity of frontal impact.

There are limitations to the present study. First, because the pregnant dummy used in this study represented 30 weeks of gestation, the obtained forces and kinematics were limited to pregnant women at approximately 30 weeks of gestation. Therefore, the results may not apply to all gestational ages of pregnant women.

Second, we performed the sled test only once or twice for each condition. However, because the sled test is a physical test using a mechanical dummy, reliable results can be obtained in a single run. In fact, an officially recognized automotive accreditation test is based on a single collision test. We therefore believe that the results of the present study are sound.

Third, we used an interior buck representing a single sedan-type vehicle in this study. The geometry of the driver’s seat does not differ appreciably among sedans, regardless of the original equipment manufacturer or class of car. However, we understand that sport utility vehicles have a different geometry from sedans with more upright sitting positions, likely resulting in different outcomes.

## 5. Conclusions

We analyzed the kinematics and applied forces of pregnant and non-pregnant women dummies sitting in the rear seat during a frontal vehicle collision. The two dummies had similar kinematics with similar forward movement in the collision. The belt loads were higher for the MAMA IIB than for the Hybrid III, because the MAMA IIB had a higher body mass than the Hybrid III. The difference in the lap belt loads were 1119 N at 29 km/h and 1981–2365 N at 48 km/h. Therefore, for restrained pregnant women with a gestational age of approximately 30 weeks sitting in the rear seat, stronger forces may apply to the lower abdomen at a higher velocity of frontal collision. Our results suggest that for restrained pregnant women sitting in the rear seat, the severity of abdominal injury and the risk of a negative fetal outcome may depend on the collision velocity. A driver should not drive at an excessive velocity when a restrained pregnant woman is sitting in the rear seat.

## Data Availability

The data presented in this study are available upon request from the corresponding author.
